# Correlation between CT volumetry and actual graft weight in living donor liver transplants in South Africa

**DOI:** 10.4102/sajr.v28i1.2917

**Published:** 2024-09-05

**Authors:** Katherine D. Calver, Owen Terreblanche, Ilonka Warnich, Francisca van der Schyff

**Affiliations:** 1 Department of Diagnostic Radiology, Faculty of Health Sciences, University of the Witwatersrand, Johannesburg, South Africa; 2 Department of Radiology, Wits Donald Gordon Medical Centre, Faculty of Health Sciences, University of the Witwatersrand, Johannesburg, South Africa; 3 Department of Diagnostic Radiology, School of Clinical Medicine, University of the Witwatersrand, Johannesburg, South Africa; 4 Department of Surgery, Wits Donald Gordon Medical Centre, Faculty of Health Sciences, University of the Witwatersrand, Johannesburg, South Africa

**Keywords:** living donor liver transplant, liver transplantation, end-stage liver disease, CT volumetry, graft weight

## Abstract

**Background:**

Liver transplantation is the definitive management for patients with end-stage liver disease. Preoperative computed tomography (CT) is used in living donor liver transplant (LDLT) for donor and graft selection as well as predicting graft weight.

**Objectives:**

The aim of this study is to establish the relationship between estimated graft volume (EGV) and actual graft weight (AGW) and ascertain a correlation coefficient that will improve the accuracy of EGV in a South African population.

**Method:**

The study included 117 LDLT between March 2013 and August 2022. Of these, 86 were left lateral (LL), 15 right lobe (R), 10 left lobe with caudate (LC), five left lobe (L) and one segment two (monosegment) grafts. Estimated graft volume and actual graft weight were compared using the Pearson coefficient and the relationship was illustrated with scatter plots.

**Results:**

Estimated graft volume and AGW had a strong positive correlation with a Pearson correlation (R) of 0.95 (*p* < 0.001). The relationship was significantly linear with a correlation coefficient of 0.71. The mean EGV was significantly higher than that of AGW (388 mL ± 249 mL vs. 353 g ± 184 g) with overestimation in 61% of cases. Left lateral and R grafts were the most prevalent LDLT graft type, both having a strong linear correlation between EGV and AGW.

**Conclusion:**

Applying a correlation coefficient of 0.71 will improve the accuracy of CT volumetry graft weight predictions.

**Contribution:**

A unique correlation coefficient will improve EGV accuracy, aiding in preoperative planning and mitigating post-operative complications in both donors and recipients.

## Introduction

The Wits Transplant unit, which forms part of the Wits Medical Centre, is the largest transplantation unit in sub-Saharan Africa and the only centre that performs Living Donor Liver transplants (LDLT) in the region. To combat the global shortage of cadaveric donors, a problem exacerbated during the COVID-19 pandemic, the Wits transplant unit makes use of split liver grafts, ABO incompatible transplants as well as running an LDLT programme. Living Donor Liver transplants allow healthy individuals (related or not) to donate part of their liver to patients with end-stage liver disease (ESLD). Despite this, in 2020, there was a 20% paediatric and 11% adult mortality rate of patients on the transplant waiting list.^1,2^

To ensure favourable transplant outcomes in LDLT, the donor needs to retain 30% - 40% of their total liver mass and the recipient needs a graft recipient weight ratio (GRWR) of at least 0.8%.^3,4,5,6,7^ This is to mitigate the risk of complications, such as small-for-size in the recipient or liver insufficiency in the donor. Both conditions arise because of insufficient liver tissue to meet the body’s metabolic and synthetic demands.^3^

Computed tomography (CT) is utilised preoperatively to determine the eligibility of potential donors and to predict liver graft weight, both being pivotal for successful outcomes in LDLT.^7,8^ The estimated graft volume (EGV) is computed using liver volumetry software and is then converted into an estimated graft weight (EGW) in a 1:1 ratio. This, however, is based on the water displacement of cirrhotic livers and therefore, does not hold true for donors of healthy liver tissue.^9^

It is well established that CT-derived EGV predicts actual graft weight (AGW) with acceptable accuracy as there is a linear correlation between the two. Many studies have sought to further improve EGV predictions of graft weight using formulas and coefficient factors.^10^ Saleem et al.^10^ found that in Egyptian living donors, 1 mL of liver volume equated to 0.96 g of liver weight, thereby making the CT volumetry predictions more accurate. Similarly, Yoneyama et al.^9^ found that the density of liver in right lobe grafts was 0.84 g/mL and 0.85 g/mL in left lobe grafts in Japanese donors.

In this study, the aim was to assess the relationship between EGV and AGW in a South African context and to test if combining the CT volumetry values with a population-specific correlation coefficient will be more accurate in predicting EGW.

## Research methods and design

### Donors

Retrospectively, all living liver donors from the Wits Donald Gordon Medical Centre, between March 2013 and August 2022, were enrolled into the study. A total of 177 donors were identified, 34 were excluded based on missing data (no preoperative CT or incomplete hospital notes), 22 patients were scanned at other hospitals (exclusion criterion), three were omitted because of difficulty in segmenting the pre-operative CT and one patient died in theatre.

### Liver volumetry

All preoperative donor CT scans were acquired on a 64-slice Phillips Ingenuity scanner using a standardised abdominal imaging protocol (arterial, early venous, late venous and delayed phases). The phases were acquired after the administration of 125 mL intravenous contrast and 20 mL of flush at 5 mL/s.

CT volumetry was performed on 3 mm slices of the early or late venous phase using the ‘liver segmentation’ add-on to the Phillips Intellispace^®^ radiology software suite. The donor livers were segmented based on the Couinaud classification into eight functional units. Each segment, numbered from one to eight, was allocated a volume in cm^3^. This volume was converted to grams based on a 1:1 ratio.

### Operative procedure and measurement of actual liver weight

Preoperative CT volumetry was used to determine the size of the graft needed for the recipient, taking donor safety into consideration. Left lateral (LL) grafts consisted of segments 2 and 3, left (L) grafts of segments 2, 3 and 4, left caudate (LC) grafts of segments 1-4 and lastly, right (R) grafts of segments 5-8.

After the donor hepatectomy was completed, the graft was taken for back table procedures, which included flushing the vessels with histidine-tryptophan-ketoglutarate (HTK) solution until the effluent was clear. The graft was then weighed on an automatic scale before being packaged and transported to the recipient’s operating theatre.

### Statistical analysis

The statistical analysis of the data was performed using IBM^®^ SPSS^®^ statistics (version 28). The results are expressed as means and standard deviations for numerical data and absolute numbers with percentages for categorical data. The ratios of EGV to body weight and AGW to body weight were compared using the paired *t* test. Pearson’s correlation coefficient was used to determine the correlation between the EGV and the AGW with results displayed using scatter plots (EGV on the *x* axis and AGW on the *y* axis). Chi-squared analysis was used in the analysis of the GRWR by graft type. A 95% confidence interval was used to assess the level of agreement between the statistical methods, and a *p* value of < 0.05 was deemed significant. Percentage deviation between the AGW and the EGV was calculated as follows (Equation 1):

$$\mathrm{Percentage}\;\mathrm{deviation}=\frac{\text{EGV}-\text{AGW}}{\text{AGW}}\times 100$$ [Eqn 1]

A positive deviation would imply overestimation and a negative deviation would imply underestimation.

### Ethical considerations

This retrospective study commenced after obtaining ethical clearance from the Human Research Ethics Committee (Medical) of the University of Witwatersrand, Johannesburg (clearance certificate no. M220739). Care was taken to protect the anonymity of the participants by allocating each a unique study number. All further documents referenced these study numbers and were stored on a password protected laptop.

## Results

**FIGURE 1:**
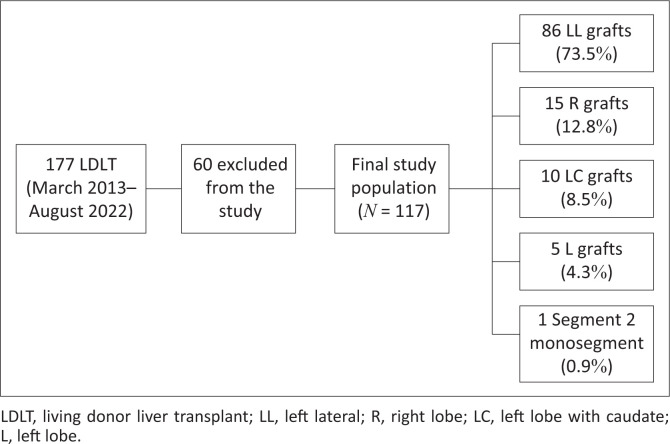
Study population and different graft types.

In total, 117 liver donors were included in the study (47 males and 70 females); the demographics are summarised in Table 1. Of all the LDLT, 83% were adult to child donations and 17% adult to adult donations. The most frequent graft type harvested was LL (73.5%) followed by R (12.8%) (Figure 1).

**TABLE 1:** Donor demographics.

Parameter	Study group		
	*n*	Mean ± s.d.	IQR
**Gender**			
Male	47	-	-
Female	70	-	-
Age (years)	-	33.4 ± 7.8	32.1–34.9
Height (m)	-	1.7 ± 0.1	1.7–1.7
Weight (kg)	-	70.4 ± 12.3	68.5–72.8
BMI (kg/m^2^)	-	24.9 ± 3.5	24.3–25.6

BMI, body mass index; IQR, interquartile range.

The EGV of the grafts averaged 388 mL ± 249 mL with a range of 127 mL – 1610 mL. The AGW averaged 353 g ± 184 g with a range of 160 g – 1300 g. The mean EGV was statistically significantly low compared to that of the AGW (*p* < 0.001).

**FIGURE 2:**
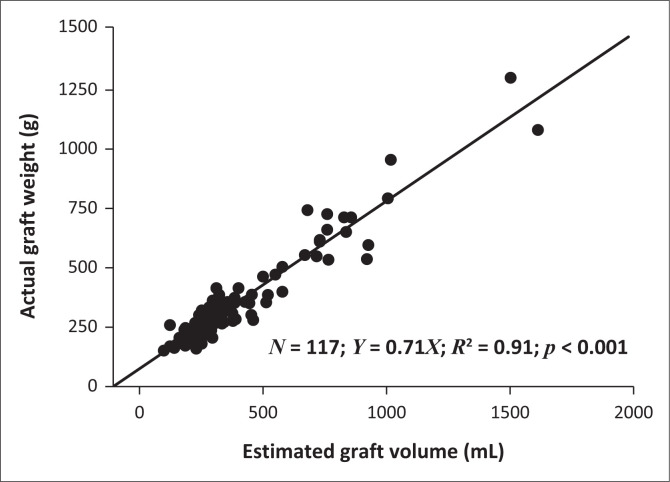
Scatter plot of actual graft weight and estimated graft volume in all living donor liver transplant grafts.

The relationship between EGV and AGW was significantly linear and statistically significant (*r* = 0.95, *p* < 0.001). The scatter plot for EGV and AGW is demonstrated in Figure 2 with a trend line equation of *y* = 0.71x, *R*^2^ = 0.91, *p* < 0.001. This implies that multiplying the CT-generated graft volume by 0.71 will more accurately estimate the graft weight. Overall, looking at percentage deviation, CT volumetry overestimated the AGW 61% of the time and underestimated the graft weight 39% of the time.

### Left lateral grafts

**FIGURE 3:**
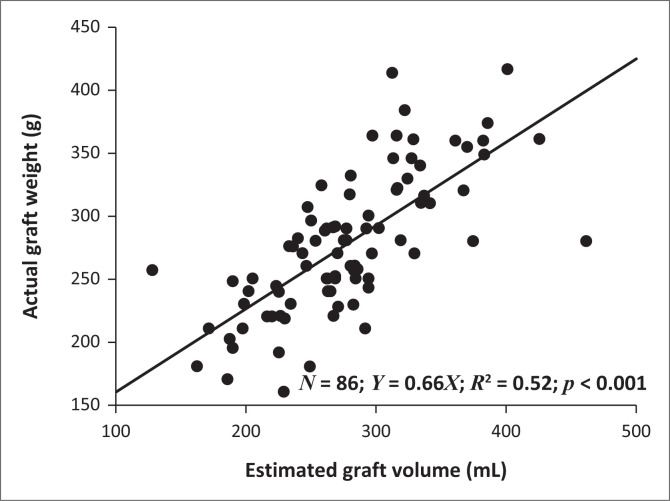
Scatter plot of actual graft weight and estimated graft volume in left lateral grafts.

The EGV mean was 278 mL ± 61.4 with a range of 127 mL – 461 mL. The AGW mean was 278 g ± 56.5 g with a range of 160 g – 417 g. There was no statistically significant difference between the EGV and AGW means (*p* = 0.92). Estimated graft volume and actual graft weight had a strong positive correlation (*Y* = 0.66x, *R*^2^ = 0.52, *p* < 0.001) illustrated in Figure 3. Computed tomography volumetry had a similar amount of overestimation as underestimation, 49% and 51%, respectively.

### Right lobe grafts

**FIGURE 4:**
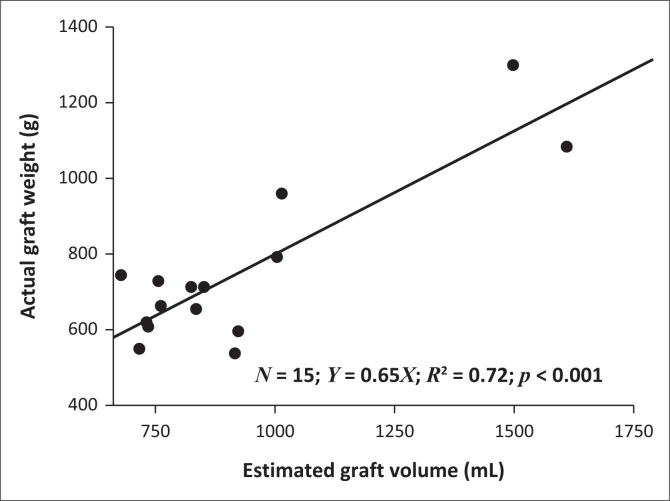
Scatter plot of actual graft weight and estimated graft volume in right lobe grafts.

The mean EGV was statistically significantly different from the AGW (925 mL ± 276 mL vs. 751 mL ± 211 mL, *p* < 0.001). Computed tomography volumetry overestimated the AGW in 93% or cases with only one underestimate. Estimated graft volume and AGW did have a positive correlation (*R* of 0.85; *p* < 0.001) that was significantly linear (*Y* = 0.65x, *R*^2^ 0.72, *p* < 0.001), as demonstrated in Figure 4.

## Discussion

Many methods of predicting graft weight have been proposed over the years; however, CT volumetry remains the gold standard. The Donald Gordon Transplant unit, like many other transplant institutions, operates under the premise that 1 mL of liver volume equates to 1 g of liver weight. Many studies have, however, shown this not to be the case, as liver tissue from healthy donors tends to have a density of less than 1 g/mL.

In this study, the liver density of healthy donors was 0.71 g/mL, and overall, CT volumetry was much more likely to overestimate the graft weight (61% frequency). Specifically looking at graft type, 100% of LC and 93% of R grafts were overestimated, whereas LL had almost equal amounts of over- and underestimation. Overestimation can be problematic in cases where the predicted graft size is borderline, and the recipient may subsequently end up with a GRWR of less than 0.8%.

Kiuchi et al.^11^ looked at the impact that graft mismatching had on graft survival. Small for size grafts, with a GRWR of < 1%, had a significantly lower graft survival than larger ones. Recipient mortality or graft loss in these cases was attributed to sepsis, graft non-function, cerebral infarction associated with immunosuppressive drugs and chronic rejection. In addition, recipients presenting in acute fulminant liver failure or advanced chronic liver disease may not have the functional reserve to cope with smaller grafts in the acute postoperative period.

Of the 117 transplants, there were four recipients (3.4%) who ended up with a GRWR of less than 0.8 (Table 2). These cases involved LL and LC grafts. In such instances, there are surgical techniques that can be used to moderate portal venous inflow such as a hemi-portocaval shunt or splenic artery ligation.^12^ These procedures mitigate the damage done by the shearing forces associated with portal hyperperfusion and provide the graft with a better chance of survival.

**TABLE 2:** The actual graft recipient weight ratio of the different graft types.

Type of graft	Variable	Actual graft recipient weight ratio	
		< 0.8	≥ 0.8
LC	Number	3.0	7.0
	Percentage (%)	30.0	70.0
LL	Number	1.0	85.0
	Percentage (%)	1.2	98.8
R	Number	0.0	15.0
	Percentage (%)	0.0	100.0

Note: *p*-value < 0.001.

LC, left lobe with caudate; LL, left lateral; R, right lobe.

Underestimation and large graft size can be problematic in paediatric recipients who weigh less than 10 kg. In this population, the native portal vein may be too small to perfuse a large graft. Large grafts also present difficulty in the primary closure of the abdominal wall post-transplant and result in raised intra-abdominal pressure.^13^ This is the so-called ‘large for size’ syndrome. Another consideration is the shape of the graft. Schukfeh et al. showed that graft weight, particularly a thicker ventral dorsal diameter, had a decreased patient survival.^13^ It is recommended that a GRWR of less than 3–3.5 is used to mitigate the risk of large for size syndrome in this patient population.^12^

Besides the conversion error of assuming 1 g/mL in healthy liver tissue, there may be several other factors that account for the discrepancies between the predicted and the AGW. These can be broadly categorised into radiological and surgical factors that affect the EGV and AGW, respectively.

The radiological factors that affect the EGV include the scanning protocol and acquisition, the software used to segment the liver and interobserver variation. Lim et al.^3^ found that CT volumetry calculated using non-contrasted versus contrasted venous phase sequences leads to less overestimation of size. Despite this, the portal venous phase is still preferred by radiologists as it better delineates the vascular and hepatic anatomy needed for segmentation. Additionally, the slice thickness also plays a role. The smaller the slice, the more accurate the volume assessment. This, however, is more time-consuming, especially if using manual volumetric techniques as there are many more slices to mark and contour.^3,14,15^ It is generally accepted that the use of 6 mm slices has adequate accuracy without taking too much time to segment.^3^ There is segmentation software that includes and others that exclude vasculature in the assessment of volume. Software that includes vessels tends to overestimate, whereas vessel-free volumetry tends to underestimate weight.^1^ Lastly, interobserver variation naturally plays a role in volume prediction in everyday practice as there are several radiologists who report on these cases.

Surgically, the transection plane of the graft may not correlate exactly to the segmentation line on CT leading to EGV/AGW mismatch. There has also been a suggestion that the preservation solution used to flush the graft may influence the graft weight.^9,14^ Histidine-tryptophan-ketoglutarate solution used by Yoneyama et al. as well as by the Wits Donald Gordon transplant unit causes graft oedema. However, given that the graft is weighed soon after being flushed, this effect is likely to be negligible.^9^

## Conclusion

In the context of LDLT in South Africa, a correlation coefficient of 0.71 multiplied by the EGV will lead to more accurate predictions of graft weight at our institution. Incorporating this into clinical practice should assist with donor and graft selection and decrease the chance of inappropriate GRWR ratios. The findings of this study are only applicable to transplants at our institution and in a South African population because of biases introduced by different CT machines and protocols, differing segmentation software, surgical protocols and population characteristics.
